# Tillage and crop establishment effects on weeds and productivity of a rice-wheat-mungbean rotation

**DOI:** 10.1016/j.fcr.2022.108577

**Published:** 2022-08-01

**Authors:** J.S. Mishra, Rakesh Kumar, Surajit Mondal, S.P. Poonia, K.K. Rao, Rachana Dubey, Rohan Kumar Raman, S.K. Dwivedi, Rahul Kumar, Kirti Saurabh, Md Monobrullah, Santosh Kumar, B.P. Bhatt, R.K. Malik, Virender Kumar, Andrew McDonald, S. Bhaskar

**Affiliations:** a ICAR Research Complex for Eastern Region, Patna, Bihar, India; b Cereal Systems Initiative for South Asia (CSISA)-CIMMYT, Patna, India; c Birsa Agricultural University, Ranchi, Jharkhand, India; d International Rice Research Institute, Los Banos, The Philippines; e Soil and Crop Sciences Section, School of Integrative Plant Sciences, Cornell University, Ithaca, NY, USA; f Indian Council of Agricultural Research, New Delhi, India

**Keywords:** Crop residue management, Direct-seeded rice, Grain yield, Triple cropping, Weed biomass, Weed density

## Abstract

Weeds are one of the key threats in sustaining the productivity of the rice-wheat cropping system in the Indo-Gangetic Plains. The development of sound integrated weed management technologies requires knowledge of mechanisms that influence weed flora composition and weed seedbank dynamics. A long-term study was initiated in 2015 at Patna, Bihar, India to evaluate the effect of seven tillage and crop establishment methods on weed density, weed seedbank composition, and crop productivity in rice-wheat-mungbean rotation. All the treatments included zero-till mungbean after wheat. Tillage and crop establishment methods had differential effects on weed and weed seedbank composition. In rice, zero-till direct-seeded rice recorded 62% lower emergence of *Cyperus iria*, 82–90% of *Echinochloa colona,* and 81–83% of total weeds compared to tilled systems, but the system of rice and wheat intensification favoured *E. colona*. In wheat, the system of wheat intensification favoured the *Phalaris minor* and *Solanum nigrum*. Zero-till rice and wheat reduced the seedbank of *Trianthema portulacastrum* by 95%, and total weed seedbank by 62% compared to the system of rice and wheat intensification. Nearly, 72% of *C. iria* seeds, 62% of grasses, and 64% of broad-leaved weeds were in 0–15 cm soil layer. Zero-till direct-seeded rice produced a 13% lower rice grain yield than conventional puddled transplanted rice. Compared to the system of wheat intensification, zero-till wheat under triple zero-till systems produced an 11.5% higher grain yield. Managing weed seedbank is a long-term endeavour. The present study revealed that tillage and crop establishment methods influence weed density and diversity. Under zero-till rice-wheat system, rice yield decreases marginally, but the system productivity maintains due to improvement in succeeding wheat yield. This system is also helpful in reducing the weed flora density and soil weed seedbank. Regular monitoring and management of emerging pests such as armyworm (*Mythimna separata*) are, however, required. The study suggests that the adoption of triple zero-tillage can be a viable option for reducing the weed density and weed seedbank concurrently increasing the system productivity of the rice-wheat-mungbean cropping system in eastern Indo-Gangetic Plains.

## Highlights

• Effect of tillage and crop establishment on soil weed seedbank in rice-wheat-mung bean system is poorly explored.
• Direct-seeded rice (DSR)-based conservation agriculture (CA) system reduced weed density and seedbank than conventional.
• More than 60% weed seeds were placed in 0–15 cm soil depth.
• Zero-till wheat after DSR produced higher yield than conventional till wheat after puddled transplant rice.
• After five years, CA system favoured infestation of armyworm (*Mythimna separata*) in wheat.

## 1 Introduction

Sustainable intensification of rice-wheat cropping system is essential in ensuring food and nutritional security in the eastern Indo-Gangetic Plains covering the eastern parts of India (eastern Uttar Pradesh, Bihar, and West Bengal provinces), Nepal and Bangladesh. Small-holder farmers of the region depend on rice and wheat for their staple food, and also for animal feed. The current production practices in rice-wheat system (transplanting of 25–30 days old rice seedlings into puddled soil, and repeated tillage in wheat) require a large number of resources (labour, water, energy) with low resource-use efficiency (Kumar et al., 2018, Mishra et al., 2021). Besides, these traditional practices also deteriorate soil health (Mondal et al., 2020, Mondal et al., 2019), increase greenhouse gas (GHG) emissions (Kumar et al., 2018, Mishra et al., 2021), and adversely affect the productivity of post-rice crops (Kumar et al., 2018, Kumar et al., 2020).

In view of increasing water, labour and energy shortage in rice-wheat system, the alternative crop establishment technologies e.g., system of rice intensification (SRI), machine transplanted rice (MTR), wet-seeded rice, direct-seeded rice (DSR), and zero-till (ZT) with or without crop residue retention have been developed and evaluated in the eastern Indo-Gangetic Plains (Jat et al., 2014, Samal et al., 2017, Singh et al., 2020, Mishra et al., 2021). Despite several advantages of resource conservation technologies compared to conventional systems, their adoption rate by the smallholder and resource-constrained farmers in many tropical and sub-tropical regions of the developing world is very low (Bolliger, 2007, Gowing and Palmer, 2008, Affholder et al., 2010) due to limited access to, and use of external inputs (seeding machinery, herbicides and others) as well as having competing demands of crop residue for animal feed and fuel (Lal, 2007, Giller et al., 2009, Keil et al., 2015), and difficulty in weed management in absence of tillage (Giller et al., 2009, Chauhan et al., 2012).

Weeds are one of the major bottlenecks in sustaining the productivity of rice-wheat systems, especially under ZT systems because of their plasticity to adapt in response to new management practices (Sosnoski and Cardina, 2006). Weeds cause a much higher loss in DSR than transplanted rice (Rao et al., 2007, Mishra and Singh, 2012, Matloob et al., 2015). The emergence of weeds simultaneously with rice seedlings or even before, lack of puddling and standing water at the early stages of the crop to check weed emergence, and absence of rice seedling size advantage over emerging weeds intensify weed problems in DSR (Kent and Johnson, 2001, Rao et al., 2007, Kumar and Ladha, 2011, Chauhan et al., 2015) and they compete more vigorously for resources with the crop than in PTR (Khaliq and Matloob, 2011). The yield losses caused by uncontrolled weeds in dry-DSR were 85–96% under CT (Chauhan and Johnson, 2011, Singh et al., 2011) and up to 98% in ZT conditions (Singh et al., 2011). Similarly, uncontrolled weeds in wheat caused a 60.5% reduction in grain yield under CT and 70% in ZT conditions (Jain et al., 2007).

The soil weed seedbank is the major source of weeds that determines the above ground weed flora composition and density in agricultural fields. The seedbank comprises new weed seeds recently shed by the plant and older seeds already present in the soil for many years (Norris, 2007, Skuodienė et al., 2013). It has been estimated that only less than 10% of the viable weed seeds produced in a particular season germinate and develop into seedlings; the remaining seeds germinate in subsequent years depending on the seed position (Swanton et al., 2000), seed dormancy, cropping system and management practices. The maximum seed reserve has been reported in 0–5 cm soil depth and decreases with increasing soil depth (Chauhan et al., 2006b, Mishra and Singh, 2012). Soil disturbance, crop rotations and crop management practices have a strong influence on weed seedbank and species densities (Grundy et al., 2003, Koocheki et al., 2009, Hosseini et al., 2014, Chen et al., 2017, Weisberger et al., 2019). A change in tillage and crop establishment methods influences species composition by direct killing of weeds or by redistributing weed seeds in different soil depths, and by changing the soil environment and thereby affecting the weed seed germination and emergence (Samarajeewa et al., 2005, Nichols et al., 2015, Sharma et al., 2020). Tillage can stimulate some weed seeds to germinate and bury other seeds which can remain viable in the soil for many years. Many weed seedlings fail to emerge if weed seeds are placed deeply (Chauhan and Johnson, 2008). Zero-till system accumulates weed seeds at the soil surface due to lack of soil disturbance (Hoffman et al., 1998, Chauhan et al., 2006b) and favours those species that can germinate from shallow depths or from within the surface crop residue layer (Barberi, Cascio, B, 2001) or that require light to germinate (El Titi, 2003, Chauhan and Johnson, 2009a). Presence of crop residue cover on the soil surface influences weed seed germination and emergence, and weed biomass by changing the soil seedbank environment (light interception, physical barrier, soil moisture, allelopathy) (El Titi, 2003, Bilalis et al., 2003, Nichols et al., 2015). The rate of reduction in weed emergence and weed biomass also depends on the quantity and quality of crop residue (Ranaivoson et al., 2018). Weed density and biomass decreased with increasing amounts of crop residues, and more than 10 Mg ha^−1^ was needed for a significant reduction in weed emergence and weed biomass as compared to bare soil without surface residues (Ranaivoson et al., 2017, Ranaivoson et al., 2018). Retaining crop residue on the soil surface under ZT system suppresses weed seedling emergence, delays the time of emergence, and allows the crop to gain an advantage over weeds (Chauhan and Johnson, 2010). Tillage and crop residues affect the efficacy of pre-emergence herbicides (MacLaren et al., 2021). Weeds and crop residues also act as alternate hosts for insects and diseases (Mishra et al., 2019a). Therefore, a thorough knowledge of the weed density and weed seedbank dynamics under conservation tillage system is required for developing successful weed management strategies.

In conventional rice-wheat system, fields remain fallow for a period of around 75–80 days during summer after harvesting of wheat crop. Pre-monsoon rains favour most of the common upland rice weeds to germinate and produce large quantities of seeds during this period (Mishra et al., 2019b), thereby enriching soil weed seedbank and aggravating the weed problems in succeeding rice. Therefore, diversification of rice-wheat system with inclusion of summer mungbean has an enormous potential to reduce the weed seedbank by offering greater soil cover during fallow period (Rao et al., 2017), besides restoring the soil fertility, increasing profitability and nutritional security of small and marginal farmers (Kumar et al., 2018). This practice over time, can help in reducing the weed seedbank and provide long-term weed management. Weisberger et al. (2019) based on a meta-analysis reported that diversification of crop rotation reduces weed density by 49%, with a greater reduction in ZT compared to CT systems.

The development of sound weed management technologies requires knowledge of mechanisms that influence composition of weed flora and weed seedbank dynamics. Estimation of weed seedbank can indicate future weed infestation. Several studies were conducted on evaluating the performance of conservation and conventional rice-wheat system based on a single crop in eastern Indo-Gangetic Plains. Information on weed flora shift, weed seedbank dynamics, productivity and profitability as influenced by tillage, crop establishment methods and residue management in double- and triple zero-till systems is lacking. Hence, the present study was undertaken to compare the effect of different tillage intensities in rice-wheat-mungbean production system. The objectives of this study were: (1) to determine the weed flora & weed seedbank density and composition established after 5 years of rice-wheat-mungbean rotation under different tillage and crop establishment methods, and (2) to find out the effect of tillage intensities on grain yield.

## 2 Materials and methods

### 2.1 Experimental site

**Fig. 1:**
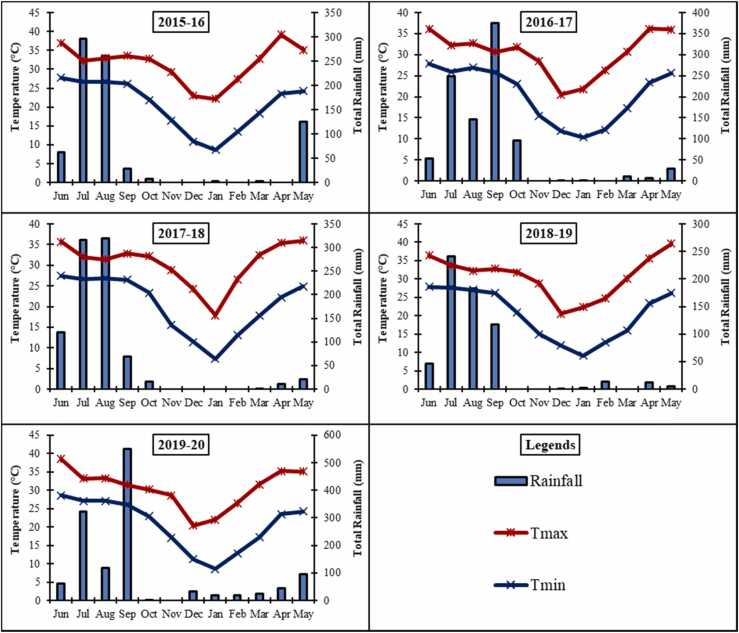
Mean monthly maximum (Tmax) and minimum (Tmin) temperatures (°C) and total precipitation (mm) during 5 cropping seasons.

A long-term experiment on tillage and crop establishment methods was establishedin rice-wheat-mungbean cropping system in June 2015 during wet season until June 2020 at the research farm of Indian Council of Agricultural Research (ICAR) – Research Complex for Eastern Region (RCER) (25°35*'* N, 85°05*'* E, and 51 m above mean sea level) Patna, Bihar, India. The climate is sub-tropical hot and humid. The soil (order Vertic Endoaqualfs) had silty loam texture (22% sand, 54% silt and 24% clay); pH 7.22; organic carbon 0.60%; electrical conductivity 0.17 dS m^−1^; available N 188 kg ha^−1^; available P 12.9 kg ha^−1^; and available K 137 kg ha^−1^. The average on-site precipitation is 1167 mm annually, of which 75–80% is received during June to September months. The average maximum temperature varies from 35.1 to 39.6°C in May and minimum temperature from 7.4 to 10.4°C in January. Mean monthly temperature and monthly precipitation of the study period (2015–16 to 2019–20) are presented in Fig. 1.

### 2.2 Treatment details and experimental design

The experimental design was a randomised complete block with three replications for rice-wheat-mungbean cropping system. Treatments consisted of seven tillage and crop establishment methods: (1) puddled random transplanted rice – broadcast wheat (RPTR–BCW); (2) puddled line transplanted rice – conventional-till wheat (LPTR–CTW; (3) puddled machine transplanted rice – zero-till wheat (CTMTR–ZTW); (4) zero-till machine transplanted rice – zero-till wheat (ZTMTR–ZTW); (5) system of rice intensification – system of wheat intensification (SRI–SWI); (6) CT direct-seeded rice – zero-till wheat (CTDSR–ZTW); and (7) zero-till DSR –- zero-till wheat (ZTDSR–ZTW). A third crop of zero-till mungbean was raised in all treatments after wheat. The individual plot size was 8.1 m × 20 m (162 m^2^). The details of tillage and crop establishment methods are presented in Table 1.

**Table 1:** Description of tillage and crop establishment (TCE), and residue management practices under rice-wheat mungbean system during five years of experimentation. [RPTR: Puddled random transplanted rice; BCW: Broadcast wheat; LPTR: Puddled line transplanted rice; CTW: Conventional-till wheat; CTMTR: CT machine transplanted rice; ZTW: Zero-till wheat; ZTMTR: Zero-till MTR; SRI: System of rice intensification; SWI: System of wheat intensification; DSR: Direct-seeded rice].

Treatment notations	Tillage			Crop establishment			Residue management		
	Rice	Wheat	Mungbean	Rice	Wheat	Mungbean	Rice	Wheat	Mungbean
T_1_: RPTR-BCW	Cultivator: 2 passes (dry tillage: DT) Rotavator: 1 pass (wet tillage: WT)	Cultivator: 2 passes Rotavator: 1 pass	Zero-till	25-days old seedlings, manually transplanted with random geometry	Broadcasting	Drill seeding with Happy Seeder	~30% incorporated in the soil	~30% retained on the soil surface	100% incorporated
T_2_: LPTR-CTW				25-days old seedlings, manually transplanted in lines at 25 × 15 cm apart.	Drill seeding with Happy Seeder				
T_3_: CTMTR-ZTW		Zero-till		18-days old seedlings, machine transplanting at 23 × 14 cm apart.			~30% retained on the soil surface		
T_4_: ZTMTR-ZTW	Zero-till (flooding before transplanting)			18-days old seedlings, machine transplanting at 23 × 14 cm apart.					100% removed
T_5_: SRI-SWI	Cultivator: 2 passes (DT) Rotavator: 1 pass (WT)	Cultivator: 2 passes Rotavator: 1 pass		12-days old seedlings, manual transplanting at 25 × 25 cm apart.	Manual seeding		~30% incorporated in the soil		100% incorporated
T_6_: CTDSR-ZTW	Cultivator: 2 passes Rotavator: 1 pass	Zero-till		Drill seeding at 22.5 cm row spacing	Drill seeding with Happy Seeder		~30% retained on the soil surface		
T_7_: ZTDSR-ZTW	Zero-till			Drill seeding at 22.5 cm row spacing					100% retained on the soil surface

Different crop residue management practices were followed for different tillage and establishment methods. 30% rice residue was either retained (for zero-till) or incorporated (for conventional till) in all treatments. Similarly, 100% mungbean residue was retained or incorporated except in ZTMTR-ZTW, where mungbean residue was removed to facilitate machine transplanting in zero-tilled soil. However, for wheat 30% residue was retained for all the treatments.

### 2.3 Agronomic management

During rainy season (June-October), rice (Cv. Arize 6444) was directly sown in rows at 22.5 cm x ~5 cm apart during 3^rd^ week of June every year by Zero-till Happy Seeder having an inclined-plate metering system (enables stubble mulching and seed drilling simultaneously; Sidhu et al., 2007) with 25 kg seed ha^–1^ at 3–4 cm seeding depth in all DSR (CT/ZT) systems. Nurseries for PTR, MTR and SRI were raised on the same day with recommended package of practice. A mat-type nursery was raised for MTR with 20 kg seed ha^−1^ (Singh et al., 2020). For PTR and SRI, nursery beds were prepared with seed rates of 15 and 7 kg ha^–1^, respectively. In SRI, 12 days old rice seedlings were uprooted from nursery and transplanted manually with single seedling at 25 × 25 cm spacing. In winter season (November-March) wheat (Cv. HD 2967) was sown during the second fortnight of November. In all CT and ZT treatments, the crop was sown in rows at 22.5 cm × ~5 cm apart with 100 kg seed ha^–1^ using Zero-till Happy Seeder, except in CT-broadcast where manual broadcasting and mixing with rotavator was done with 120 kg ha^–1^, and SWI where single seed was manually dibbled at 25 × 25 cm apart with 25 kg seed ha^–1^. During summer (April-June), short duration (60–65 days) mungbean (Cv. Samrat) was sown under zero-till conditions immediately after wheat harvest using Zero-till Happy Seeder in 22.5 cm × ~10 cm spacing with 30 kg seed ha^–1^ during second week of April. Recommended doses of 120 kg N, 60 kg P_2_O_5_ and 60 kg K_2_O ha^–1^ as urea, di-ammonium phosphate (DAP) and muriate of potash, respectively were applied to rice and wheat. The 1/3^rd^ of recommended N and the full doses of P and K were applied as basal. Remaining 2/3^rd^ N was applied in two equal splits at active tillering and panicle initiation stages. For mungbean 100 kg DAP ha^–1^ was applied as basal through Happy Seeder.

Pendimethalin (30% EC) at 1.0 kg a.i. ha^–1^ in DSR and pretilachlor at 0.75 kg a.i. ha^–1^ in TPR were applied as pre-emergence (2 DAS/DAT), and bispyribac-sodium at 25 g a.i. ha^–1^ as post-emergence was applied at 20 DAS/DAT. In wheat ready-mix combination of sulfosulfuron (75%WG) + metsulfuron methyl (5%WG) @ 32 (30 +2) g a.i. ha^–1^ was applied as post-emergence (25 days after sowing). For weed control in mungbean, pendimethalin at 1.0 kg a.i. ha^–1^ was applied as pre-emergence (next day after seeding). A knapsack sprayer fitted with a flat-fan nozzle with 500 L ha^–1^ of water was used for applying herbicides. An untreated area (2 m × 2 m in each plot) was kept to assess the weed infestation. A bund (6 in. in height) was prepared for 2 m × 2 m area and covered by a polythene sheet during herbicide applications. Rice, being a rainy season crop, was irrigated with flood irrigation (~5 cm water depth) once hairline cracks appear depending on the occurrence of dry spells during the cropping season. On average, 4–7 irrigations were applied in rice per year based on rainfall. No separate irrigation method was followed for the SRI system. In wheat, flood irrigations (~5 cm water depth) were applied at critical growth stages (CRI, tillering, flowering and grain filling stages). In mungbean, in addition to pre-sowing irrigation, two irrigations (at 25 and 45 days after sowing) were applied. Irrigation was applied through polyvinyl chloride pipes of 10-cm diameter, and in each irrigation ~5-cm water was applied. The amount of irrigation water applied to each plot was measured using a water metre. After picking its matured pods, plants were retained in ZTDSR and desiccated with spray of paraquat (Gramoxone 24% SL) at 0.48 kg a.i. ha^–1^ before rice seeding; removed in ZTMTR to facilitate mechanical transplanting of rice; and ploughed down in CTDSR/PTR/SRI treatments.

### 2.4 Rice equivalent yield

The productivity of rice-wheat-mungbean cropping system was compared by converting the grain yields of wheat and mungbean into rice equivalent yield (REY Mg ha^–1^). The system productivity was calculated as the sum of rice yield and REYs of wheat and mungbean for each treatment.

(1) REY of wheat = [(Wheat grain yield × MSP of wheat) / (MSP of rice)]

(2) REY of mungbean = [(Mungbean grain yield × MSP of mungbean) / (MSP of rice)]

(3) System productivity = rice grain yield + REY of wheat + REY of mungbean

Where, MSP is Minimum support price of Govt. of India.

### 2.5 Weed flora and weed seedbank analysis

Weed density (number m^–2^) was recorded from untreated area (2 m × 2 m in each plot) to assess the weed infestation. Weed count (species wise and total), for estimating weed density and their composition, were recorded each year with the help of a quadrate (0.5 m × 0.5 m) placed randomly at four places in each plot. Weed density in rice could not be recorded in the first year (2015). Weed count was recorded at 30 days after sowing (DAS)/days after transplanting (DAT) in rice and at 60 DAS in wheat. To record weed dry weight at 75 DAS/DAT, weeds were cut at ground level, washed with tap water, sun-dried, hot-air oven-dried at 70 ℃ for 48 h, and then weighed.

The weed seedbank studies were undertaken at the end of 4^th^ year rotation by the ‘seedling emergence’ method. The soil weed seedbank density and composition were estimated by the ‘seedling emergence’ method as described by Koocheki et al. (2009) and MacLaren et al. (2021). Although this method is time consuming, and underestimates the absolute weed seedbank size, it provides a more accurate estimation of species composition than the seed extraction method (Cardina and Sparrow, 1996). Sampling of weed seedbank was done in mid-June 2019 after harvest of mungbean crop (after completion of the fourth crop rotation). Soil samples were taken using a 4 cm diameter metal core from two depths, 0–15 and 15–30 cm, from five areas in each plot. A total of 210 soil cores “5 samples by 2 depths by 7 TCE methods by 3 replications” were taken from the whole experiment. All samples for a given depth were bulked to make a composite soil sample per plot. Bulked soil samples were partially air-dried and then any clods broken by hand. Soil debris and large root fragments were separated from the soil samples. One kg soil samples for each depth per plot were prepared and spread on 40.4 × 30.3 × 9.5 cm plastic trays with ~2 cm soil layer thickness. Subsequently, these trays were placed in a greenhouse and watered to keep the soil at field capacity. The emerged weed seedlings were identified, counted, and removed until emergence was nil. Soil was then dried, rewatered, and stirred to initiate further emergence. This cycle was repeated approximately monthly from July to December 2019. Some of the weed seedlings such as *Trianthema portulacastrum* and *Cyperus iria* were identified to species level. Because of the morphological similarity among grassy weeds at initial stages, these were grouped as ‘total grasses’. However, the total proportion of broad-leaved weeds also consisted of *T. portulacastrum* in addition to other unidentified broad-leaved weeds. Estimation of the vertical distribution of the weed seeds was made from the number of seedlings that emerged from the soil cores of different depths (Mishra and Singh, 2012).

### 2.6 Statistical analysis

Data on yield, and weeds were subjected to analysis of variance (ANOVA) following a randomized complete block design (RCBD). Duncan’s Multiple Range Test (DMRT) test was used for comparisons of means among the treatments at p < 0.05 using Statistix 8.1 statistical package (Gomez and Gomez, 1984). The normality of square-root transformed data was checked by the Shapiro-Wilk test and found to be normally distributed.

## 3 Results

### 3.1 Weed density and weed biomass

#### 3.1.1 Weeds in rice

Irrespective of crop establishment methods and years, the major weeds associated with rice were awnless barnyard grass [*Echinochloa colona* (L.) Link], Chinese sprangletop [*Leptochloa chinensis* (L.) Nees.], rice flat sedge (*Cyperus iria* L.), horse purselane (*Traianthema portulacastrum* L.), day flower (*Commelina benghalensis* L.), water primerose (*Ludwigia parviflora* Roxb.), pink node flower (*Caesulia axillaris* Roxb.) and blistering ammania (*Ammania multiflora* Roxb.) in varying density. Bermuda grass [*Cynodon dactylon* (L.) Pers.] was noticed in ZTMTR-based system after 3^rd^ cropping cycle. Tillage and crop establishment methods significantly (P < 0.05) influenced total weed density in rice. Total weed density was reduced drastically (P < 0.05) during 2017 and 2018 compared to 2016. However, it started increasing again in 2019. Among different crop establishment methods, triple ZT-based establishment method (ZTDSR-ZTW) had a significantly (P < 0.05) lower weed density, which was 81% lower than that of conventional-till machine transplanted (CTMTR) system in 2016. In 5^th^ year (2019), mechanical transplant rice (CT/ZT) had a significantly (P < 0.05) higher total weed density (150–169 m^–2^) followed by puddle transplanting (93–99 m^–2^), SRI (51 m^–2^) and CTDSR (38 m^–2^). On five years mean basis, the lowest density (24.9 m^–2^) was recorded with ZTDSR-ZTW with crop residue retention on soil surface. Tillage and crop establishment systems significantly (P < 0.05) affected total weed dry weight in rice at 75 DAS/DAT. The year x treatment interaction for weed density was significant (P < 0.001). Maximum dry biomass (420 g m^–2^) was recorded with ZTMTR-ZTW system followed by ZTDSR, CTMTR, SRI and CTDSR - based production systems (133.7–173.3 g m^–2^), which were on a par with each other. Puddled transplanted rice-based system (RPTR/LPTR) recorded significantly (P < 0.05) lower weed biomass (30.7–83.5 g m^–2^) (Fig. 3).

Maximum emergence of *C. iria* was noticed during 2016, which decreased drastically in 2017 and 2018, but again started increasing in 2019 (Table 2). Different TCE methods significantly (P < 0.05) influenced the emergence of *C. iria*. In 2016, the maximum weed density (192 m^–2^) was recorded with CTMTR-ZTW system followed by RPTR-broadcast wheat (129 m^–2^) and SRI-SWI (118 m^–2^) systems. Rice under ZTDSR had the lowest weed emergence (33 m^–2^) followed by ZTMTR (64 m^–2^). ZT system (ZTDSR/ZTMTR-ZTW) recorded a 62% lower emergence of *C. iria* as compared to tilled system. In the 5^th^ year (2019), the emergence of *C. iria* was significantly (P < 0.05) higher in mechanical transplanted rice (CTMTR/ZTMTR)-based system compared to other establishment methods. Conventional methods of rice and wheat establishment (PTR-CTW) had a higher density of *T. potulacastrum* compared to other methods in 2016. However, its emergence was almost nil in successive years, except in 2017 when its density was drastically higher in CTDSR (120 m^–2^) compared to ZTDSR (11 m^–2^). Significantly (P < 0.05) higher density of *E. colona* was observed in SRI-SWI-ZTM system, irrespective of years. Its overall density was low during initial years but increased in 2019. DSR (ZT/CT) resulted in a significant reduction in density of *E. colona* by 82% and 90% as compared to PTR and SRI systems, respectively. Density of *L. parviflora* was also very low till 2018. Significantly (P < 0.05) the lowest emergence of *L. parviflora* was noted in ZTDSR-ZTW. It was completely eliminated in the 5^th^ year.

**Table 2:** Weed density in rice under different tillage and crop establishment methods in rice-wheat-mungbean system; mean values followed by different lower case letters within a column and different upper case letters within a row or column are significantly different at P < 0.05. [RPTR: Puddled random transplanted rice; BCW: Broadcast wheat; LPTR: Puddled line transplanted rice; CTW: Conventional-till wheat; CTMTR: CT machine transplanted rice; ZTW: Zero-till wheat; ZTMTR: Zero-till MTR; SRI: System of rice intensification; SWI: System of wheat intensification; DSR: Direct-seeded rice].

Treatments	*Cyperus iria* (no. m^−2^)					*Trianthema portulacastrum* (no. m^−2^)				
	2016	2017	2018	2019	Mean	2016	2017	2018	2019	Mean
RPTR-BCW	129b	3.33b	3.00b	29.67b	41.3BC	36a	0.0	0.0	1.5b	9.4B
LPTR- CTW	95bc	3.33b	3.17b	56.67ab	39.5 C	21b	0.0	0.0	0.0c	5.3 C
CTMTR-ZTW	192a	2.67b	2.00b	101.67a	74.6 A	4c	0.0	0.0	2.17a	1.5E
ZTMTR-ZTW	64 cd	6.33a	4.67a	103a	44.5B	2c	0.0	0.0	0.67c	0.7E
SRI-SWI	118b	2.67b	2.33b	4.25b	31.8D	2c	0.0	0.0	0.25c	0.6E
CTDSR-ZTW	98bc	0.33c	0.5c	20.33b	29.8D	0c	120a	0.0	0.42c	30.1 A
ZTDSR-ZTW	33d	0.0c	0.0c	16.17b	12.3E	3c	11b	0.0	0.75c	3.7D
Mean	104.0 A	2.7 C	2.2 C	47.4B		9.7B	18.7 A	0.0D	0.82 C	
P-value (Year*Treatment)	< 0.001					< 0.001				
	*Echinochloa colona* (no. m^−2^)					*Ludwigia parviflora* (no. m^−2^)				
RPTR-BCW	2.0b	2.33b	1.83d	22.67ab	7.2B	1.3d	0.33c	0.5bc	30.75ab	8.2B
LPTR- CTW	2.0b	2.67b	2.00d	7.83abc	3.6D	0.8d	1.0c	0.67bc	19abc	5.4 C
CTMTR-ZTW	1.0b	5.67ab	5.17b	10.25abc	5.5 C	5.2b	3.0b	2.5b	32.92a	10.9 A
ZTMTR-ZTW	0.0b	4.33b	4.33bc	19.67abc	7.1B	7.2a	3.0b	2.33bc	11.42abc	6.0 C
SRI-SWI	12.0a	8.33a	8.00a	27.00a	13.8 A	1.7d	6.67a	6.67a	8.83abc	6.0 C
CTDSR-ZTW	3.0b	3.67b	3.00 cd	4.17bc	3.5D	5.1b	1.67bc	0.0c	3.92bc	2.7D
ZTDSR-ZTW	5.0b	3.0b	2.67d	1.25c	3.0D	3.55c	0.0c	1.83bc	1.58c	1.7E
Mean	3.6 C	4.3B	3.9 C	13.3 A		3.6B	2.2 C	2.1 C	15.5 A	
P-value (Year*Treatment)	< 0.001					< 0.001				
	*Commelina benghalensis* (no. m^−2^)					Total weed density (no. m^−2^)				
RPTR-BCW	2.0b	3.0a	1.83b	0.0	1.7B	182b	9.32c	7.17dfe	98.58ab	74.3B
LPTR- CTW	6.0a	2.0b	2.67a	0.0	2.7 A	132c	10.0bc	9.00 cd	93.17ab	61.0 C
CTMTR-ZTW	1.0b	1.33c	0.83 cd	0.0	0.8 C	250a	15.67b	11.33bc	169.17a	111.5 A
ZTMTR-ZTW	0.0b	2.0b	1.17c	0.0	0.8 C	138c	18.99b	13.5b	150.08a	80.1B
SRI-SWI	0.0b	1.33c	1.00 cd	0.0	0.6D	149c	25.67b	18.00a	51.08b	60.9 C
CTDSR-ZTW	0.0b	0.67d	0.50de	0.0	0.3E	152c	128.01a	4.00e	38.17b	80.6B
ZTDSR-ZTW	1.0b	0.0e	0.0e	0.0	0.3E	48d	16.33b	6.17de	29.0b	24.9D
Mean	1.4 A	1.5 A	1.1B	0 C		150.0 A	32.0 C	9.9D	89.9B	
P-value (Year*Treatment)	< 0.001					< 0.001				

#### 3.1.2 Weeds in wheat

Irrespective of crop establishment methods and years, major weeds associated with wheat were little seed canary grass (*Phalaris minor* Retz*.*), strawberry clover (*Trifolium fragiferum* L.), common lambsquarter (*Chenopodium album* L.), common vetch (*Vicia sativa* L.), black nightshade (*Solanum nigrum* L.) and toothed dock (*Rumex dentatus* L.) in varying density. Bermuda grass [*Cynodon dactylon* (L.) Pers.] was noticed in ZTMTR-based system after 3^rd^ cropping cycle. The total weed density in wheat increased progressively up to 2^nd^ year in all the tillage and crop establishment methods, and thereafter no definite trend was observed. In ZTW preceded by DSR or MTR, the increase was up to 4^th^ year, whereas in case of SWI and CTW, the weed density increased till 3^rd^ year only. The lowest weed density during first two years was recorded with ZTW preceded either by DSR/MTR systems. ZTDSR-ZTW recorded the lowest total weed in all the years, except in 2017–18, where MTR based ZTW recorded the lowest density of total weeds. The year × treatment interaction for weed density was significant (P < 0.01). The DSR-based ZTW system produced significantly (P < 0.05) lower weed biomass (18–20 g m^–2^) compared to conventional till-based production system systems (30–73 g m^–2^). Maximum weed dry weight (73 g m^–2^) in wheat was recorded with SRI-SWI system (Fig. 4).

Density of *P. minor* was significantly influenced by crop establishment methods. In 2015–16, zero-till wheat after DSR or MTR recorded the lower *P. minor* density compared to conventional till wheat grown after PTR/SRI (Table 3). ZT wheat after ZTDSR/ CTDSR/ ZTMTR reduced density of *P. minor* by 50%, 62% and 78%, respectively compared to CTW, broadcast and SWI systems. Almost similar trend was observed during 2016–17. However, in 3^rd^ year (2017–18), trend was reversed and a significantly higher density of *P. minor* (54.7–58.0 m^–2^) was noted in ZT wheat preceded by DSR compared to broadcast/ CTW/SWI (4.7–14.7 m^–2^) preceded by PTR and SRI systems. The overall density of *P. minor* increased in the 4^th^ year compared to previous three years. Significantly maximum seedling emergence of *P. minor* was recorded with ZTW preceded by ZTMTR. Broadcast being on a par with CTW recorded the lowest emergence of *P. minor*. In the 5^th^ year, density of *P. minor* was drastically reduced, irrespective of the TCE methods.

**Table 3:** Weed density in wheat under different tillage and crop establishment methods in rice-wheat-mungbean system; mean values followed by different lower case letters within a column and different upper case letters within a row or column are significantly different at P < 0.05. [RPTR: Puddled random transplanted rice; BCW: Broadcast wheat; LPTR: Puddled line transplanted rice; CTW: Conventional-till wheat; CTMTR: CT machine transplanted rice; ZTW: Zero-till wheat; ZTMTR: Zero-till MTR; SRI: System of rice intensification; SWI: System of wheat intensification; DSR: Direct-seeded rice].

Treatments	Year											
	2015–16	2016–17	2017–18	2018–19	2019–20	Mean	2015–16	2016–17	2017–18	2018–19	2019–20	Mean
	*Phalaris minor* (no. m^−2^)						*Trifolium fragiferum* (no. m^−2^)					
RPTR-BCW	42.0b	54.3a	4.7c	15.7c	0.0	23.3D	12.7b	11.3d	32.3b	1.3a	0.0	11.5 C
LPTR- CTW	32.3bc	43.3b	13.0c	39.7c	3. 7a	26.4D	3.3b	50.7a	56.3a	7.3a	0.0	23.5B
CTMTR-ZTW	23.0c	28.0c	7.3c	63.7b	2. 7a	24.9D	4.3b	42.7ab	10.7c	0.3a	0.0	11.6 C
ZTMTR-ZTW	16.0c	23.7d	27.7b	162.7a	8. 7a	47.8 A	10.7b	28.0c	6.3c	0.7a	0.0	9.1D
SRI-SWI	71.7a	52.3a	14.7c	68.3b	11.3a	43.7AB	41.7a	37.3bc	53.0a	10.3a	0.0	28.5 A
CTDSR-ZTW	16.3c	24.7d	58.0a	88.0b	10. 7a	39.5BC	7.7b	13.7d	5.7c	2.0a	0.0	5.8E
ZTDSR-ZTW	15.7c	32.7c	54.7a	83.0b	5.0a	38.2 C	10.3b	12.7d	10.3c	0.0	0.0	6.7E
Mean	31.0 C	37.0B	25.7D	74.4 A	6.0E		13.0 C	28.1 A	24.9B	3.1D	0.0E	
P-value (Y*T)	< 0.01						< 0.01					
	*Solanum nigrum* (no. m^−2^)						*Rumex dentatus* (no. m^−2^)					
RPTR-BCW	1.3c	21.7b	36.7b	10.67b	11.0b	16.3C	4.7b	7.3a	0.0	0.0	0.0	2.4B
LPTR- CTW	2.0b	19.0b	114.0a	19.0b	11. 7b	33.1B	1.3b	7.7a	6.3a	0.0	0.0	3.1 A
CTMTR-ZTW	0.7c	15.3b	14.3c	14.67b	5.0b	10.0D	0.0	6.0a	1.0c	0.0	0.0	1.4 C
ZTMTR-ZTW	0.7c	1.7c	15.3c	4.33b	4. 7b	5.4E	1.7b	0.0	0.0	0.0	0.0	0.3E
SRI-SWI	5.3a	46.3a	142.0a	67.67a	26.0a	57.5 A	8.3a	6.7a	1.0c	0.0	0.0	3.2 A
CTDSR-ZTW	0.3c	0.0	6.7c	4.67b	10.0b	4.3E	3.3b	0.0	1.0c	0.0	0.0	0.9D
ZTDSR-ZTW	0.3c	0.0	9.3c	6.0b	4.7b	4.1E	1.3b	6.3a	3.3b	0.0	0.0	2.2B
Mean	1.5E	14.9 C	48.3A	18.1B	10.4D		2.9B	4.9 A	1.8 C	0.0D	0.0D	
P-value (Y*T)	< 0.01						< 0.01					
	*Chenopodium album* (no. m^−2^)						Total weed density (no. m^−2^)					
RPTR-BCW	2.3b	5.7ab	2.7c	3.67c	0.0	2.9D	68.0b	102.3bc	83.4b	31.7c	13.0c	59.7D
LPTR- CTW	2.0b	3.7c	6.0ab	9.33b	1.3	4.5B	46.7bc	124.4b	202.6a	80.7b	19.0c	94.7B
CTMTR-ZTW	1.7b	5.0b	5.0b	2.33c	0.0	2.8D	37.0c	97.0c	40.3c	85.3b	13. 7c	54.7D
ZTMTR-ZTW	0.7b	7.0a	8.0a	1.67c	0.0	3.5 C	39.7c	61.4d	65.3bc	171.0a	22.0b	71.9 C
SRI-SWI	7.7a	5.7ab	5.7ab	18.67a	2.3	8.0 A	139.3a	156.3a	219.4a	169.3a	43. 7a	145.6 A
CTDSR-ZTW	1.7b	3.0c	1.3c	3.67c	0.3	2.0E	31.7c	41.4d	76.7b	100.0b	26.0b	55.2D
ZTDSR-ZTW	1.0b	4.3c	1.0c	4.33c	2.3	2.6D	36.3c	63.0d	83.6b	94.3b	15.3c	58.5D
Mean	2.4D	4.9B	4.2 C	6.2 A	0.9E		57.0D	92.3 C	110.2 A	104.6B	21.8E	
P-value (Y*T)	< 0.01						< 0.01					

Irrespective of tillage and crop establishment methods, seedling emergence of *T. fragiferum* increased in the second year compared to initial year, but started declining thereafter, and was completely eliminated in the 5^th^ year. Significantly higher seedling emergence was recorded with SWI system compared to other TCE methods. However, in 2/3^rd^ year, maximum emergence was noted with CTW preceded by TPR. ZTW after DSR/ZTMTR resulted in lower emergence of this weed compared to conventional systems. In general, density of *S. nigrum* was very low in first year, increased progressively until 3^rd^ year and started declining thereafter (Table 3). Irrespective of the year, SWI had the maximum density of *S. nigrum*. ZTW preceded by DSR or MTR resulted in 70%, 100%, 89%, 64%, and 35% decline in *S. nigrum* density compared to conventional till wheat (BCW/CTW) preceded by PTR, and 94%, 100%, 94%, 92% and 72% compared to SWI preceded by SRI, during 2015–16, 2016–17, 2017–18, 2018–19 and 2019–20, respectively. Density of *R. dentatus* was comparatively low. It was completely eliminated after third year of cropping. Significantly higher emergence was recorded with SWI system in the first year. In general, density of *C. album* increased until 4^th^ year and decreased in 5^th^ year. ZTW after DSR production system resulted in a significant reduction in its emergence compared to SWI after SRI system. Percent reduction in emergence of *C. album* varied from 43% in 5^th^ year to 82% in first year.

#### 3.1.3 Weed seedbank of the system

Among the broad-leaved weeds, *T. portulacastrum* was dominant. *C. iria* was the only sedge observed in the soil seedbank. Irrespective of the treatments, major portion (46%) of weed seedbank was comprised of broad-leaved weeds (Table 4) followed by sedges (31%), and grasses (23%). TCE methods did not influence soil seedbank of sedges and grasses, but significantly (P < 0.05) influenced the broad-leaved weeds (BLW) and the total weeds. Significantly higher weed seedbank density of *T. portulacastrum*, total BLW, and total weeds were recorded with SRI-SWI-ZTM system, where soil was disturbed during both rice and wheat seasons. Tillage systems disperse weed seeds throughout tillage profile. Triple zero-tilled production system (ZTDSR-ZTW) drastically reduced seedbank of *T. portulacastrum* by 95% and total weed seedbank by 62% as compared to SRI-SWI-ZTM system (Table 4).

**Table 4:** Effect of tillage and crop establishment methods on soil weed seedbank (no. kg^−1^ soil); mean values followed by different lower case letters within a column or row are significantly different at P < 0.05. [RPTR: Puddled random transplanted rice; BCW: Broadcast wheat; LPTR: Puddled line transplanted rice; CTW: Conventional-till wheat; CTMTR: CT machine transplanted rice; ZTW: Zero-till wheat; ZTMTR: Zero-till MTR; SRI: System of rice intensification; SWI: System of wheat intensification; DSR: Direct-seeded rice].

Treatments	Soil layer (cm)														
	0–15	15–30	Mean	0–15	15–30	Mean	0–15	15–30	Mean	0–15	15–30	Mean	0–15	15–30	Mean
	*C. iria*			Total grassy weeds			*T. portulacastrum*			Total broad-leaved weeds			Total weeds		
RPTR-BCW	17.69	5.00	11.33a	6.00	10.67	8.33a	10.33	5.00	7.67b	24.0	14.0	19.0b	48.0	29.67	38.67b
LPTR- CTW	12.67	5.67	9.17a	9.00	6.00	7.50a	6.00	6.33	6.17b	18.33	15.0	16.67b	40.0	26.67	33.33b
CTMTR-ZTW	21.67	11.00	16.33a	20.00	4.33	12.17a	2.67	1.67	2.17b	17.33	12.67	15.0b	59.0	28.00	43.5b
ZTMTR-ZTW	17.33	4.67	11.00a	14.00	7.67	10.83a	3.33	5.00	4.17b	14.0	12.33	13.17b	45.33	24.67	35.0b
SRI-SWI	19.67	10.33	15.00a	11.17	7.67	9.67a	36.67	11.00	23.86a	72.33	22.67	47.5a	104.0	40.67	72.17a
CTDSR-ZTW	13.00	9.00	11.00a	8.33	5.67	7.00a	4.67	1.00	2.83b	12.33	10.0	11.17b	33.67	24.67	29.17b
ZTDSR-ZTW	24.00	3.33	13.67a	9.33	5.67	7.50a	0.67	1.67	1.17b	6.00	6.33	6.17b	39.33	15.33	27.33b
Mean	18.00a	7.00b	–	11.19a	6.81b	–	9.19a	4.52b	–	23.48a	13.29b	–	52.67a	27.1b	–

**Fig. 2:**
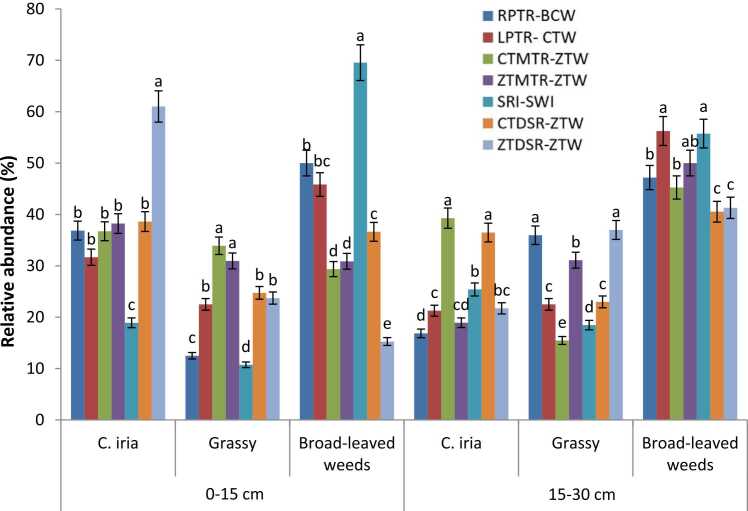
Relative abundance of key weed species in different depths (0–15 and 15–30 cm) under various tillage and crop establishment methods. Vertical bars represent the standard error of the mean; bars followed by different lower-case letters are significantly different at P < 0.05. [RPTR: Puddled random transplanted rice; BCW: Broadcast wheat; LPTR: Puddled line transplanted rice; CTW: Conventional-till wheat; CTMTR: CT machine transplanted rice; ZTW: Zero-till wheat; ZTMTR: Zero-till MTR; SRI: System of rice intensification; SWI: System of wheat intensification; DSR: Direct-seeded rice].

Tillage and crop establishment (TCE) methods had a significant (P < 0.05) influence on relative abundance of weeds in soil seedbank (Fig. 2). Weed seed numbers declined significantly (P < 0.05) as sampling depth increased in all TCE treatments (Table 4). In the present study, ~72% of *C. iria* seeds, 62% of grasses, 64% of broad-leaved weeds and 66% of total weed seeds were placed in 0–15 cm soil layer. The placement of weed seeds in varying soil depths was influenced by TCE methods. Relatively higher proportion (79–88%) of *C. iria* seeds were concentrated in 0–15 cm soil depth in undisturbed TCE system (ZTMTR-ZTW and ZTDSR-ZTW) compared to 65–69% in conventional production system (Table 4).

### 3.2 Yield and system productivity

Rice grain yield was significantly (P < 0.05) affected by TCE methods. It ranged from 4.86 Mg ha^–1^ in ZTMTR-ZTW to 6.27 Mg ha^–1^ in LPTR-CTW (Tables 5 & S1). Conventional puddled transplanted rice (LPTR and RPTR) yielded at par with SRI and ZTDSR but was significantly superior to other TCE methods. Rice yield in conventional PTR production system was significantly (P < 0.05) higher by 16.56% compared to DSR, and 23.57% compared to MTR. Wheat grain yield (4.83 Mg ha^−1^) under ZTDSR-ZTW was at par with that of CTDSR-ZTW and conventional system, but significantly higher by 11.5% over SRI-SWI-ZTM (4.33 Mg ha^−1^) systems. The conventional till drill-sown wheat (CTW) produced a slightly higher (5%) yield than the broadcast sowing (BCW) (Table5). In the present study, CT wheat yield after puddled rice was at par with ZT wheat after DSR. Mungbean grain yield in the 5^th^ year was quite low, irrespective of treatments due to frequent excess rains during cropping period (Fig. 1) resulting in poor seed setting, despite vigorous crop growth. Moreover, due to frequent rains, second pod picking which contributes ~30% to grain yield, could not be done. Grain yield of mungbean ranged from 0.45 Mg ha^–1^ in RPTR-BCW to 0.64 Mg ha^–1^ in ZTMTR-ZTW (Table 5). There was no definite trend observed in mungbean productivity due to TCE methods. As mungbean seed was drilled uniformly in anchored wheat stubbles under all treatments, differences in yield among the treatments were marginal. Tillage and crop establishment methods significantly influenced system productivity (Table 5). System productivity in terms of rice-equivalent yield (REY) ranged from 12.09 Mg ha^–1^ in ZTMTR-ZTW to 13.25 Mg ha^–1^ under LPTR-CTW. System productivity in zero-till production system (ZTDSR-ZTW) was on a par with conventional system (LPTR-CTW).

**Table 5:** Effect of tillage and crop establishment methods on crop yields in the 5th cropping season; mean values followed by different lower case letters within a column are significantly different at P < 0.05. [RPTR: Puddled random transplanted rice; BCW: Broadcast wheat; LPTR: Puddled line transplanted rice; CTW: Conventional-till wheat; CTMTR: CT machine transplanted rice; ZTW: Zero-till wheat; ZTMTR: Zero-till MTR; SRI: System of rice intensification; SWI: System of wheat intensification; DSR: Direct-seeded rice].

Treatment	Crop yields (Mg ha^−1^)			System productivity (REY, Mg ha^−1^)
	Rice	Wheat	Mungbean	
RPTR-BCW	6.26a	4.44ab	0.45c	12.73ab
LPTR- CTW	6.27a	4.66ab	0.52abc	13.25a
CTMTR-ZTW	5.28bc	4.72a	0.47c	12.12b
ZTMTR-ZTW	4.86c	4.43ab	0.64a	12.09b
SRI-SWI	6.04ab	4.33b	0.57abc	12.88ab
CTDSR-ZTW	5.29bc	4.71a	0.62ab	12.38ab
ZTDSR-ZTW	5.46abc	4.83a	0.47bc	12.46ab

## 4 Discussion

### 4.1 Weed density and weed biomass

The present study indicated that changes in tillage and crop establishment methods in rice-wheat-mungbean cropping system significantly (P < 0.05) affected density and diversity of weed species. Different TCE methods significantly influenced emergence of *C. iria*. Chauhan and Johnson (2009a) reported that germination of *C. iria*, *C. difformis* and *Fimbristylis miliacea* (L.) Vahl. is stimulated by light and warm fluctuating temperature. Zero-tilled production systems (ZT and residue cover) might have restricted solar radiation to penetrate the soil compared to CT system, resulting in reduced germination of *C. iria*. The density of *T. portulacastrum* in 2016 was significantly (P < 0.05) higher in puddle transplanted rice (RPTR and LPTR) compared to other methods of rice establishment. Puddling in transplanted rice might have brought *T. portulacastrum* seeds to soil surface from deeper soil. However, in subsequent years, the weed density become negligible in all the treatments, except in 2017, where drastically higher density was noted in CTDSR (120 m^–2^) as compared to ZTDSR (11 m^–2^) (Table 2). Shallow tillage and soil incorporation of mungbean residues in CTDSR might have exposed the weed seeds to solar radiation that stimulates weed seed germination, resulting in higher emergence contrary to ZTDSR where mungbean crop residues inhibited its emergence due to mulching effect. Significantly higher density of *E. colona* in SRI-based system in all the years was probably due to favourable conditions (wider spacing and moist soil conditions). The lower emergence of *E. colona* in DSR based production system might be due to fact that most *E. colona* seeds remained on soil surface due to less soil disturbance and were predated by birds and other insects. Accumulation of crop residue mulch on soil surface might have acted as a physical barrier in emergence of *E. colona* seedlings. Our results are in contrast to many other reports that concluded higher density of *E. colona* in DSR (Singh et al., 2005, Chauhan and Johnson, 2009b). Aquatic environment maintained under puddling and transplanting system of rice establishment favoured emergence of *L. parviflora* (Wagner et al., 2007, Hussner, 2012), increasing density by 88% as compared to unpuddled DSR system. Density of *Commelina benghalensis* was very less irrespective of the year (0–1.5 no. m^−2^) and crop establishment method (0.3–2.7 no. m^−2^).

Soil disturbance, soil characteristics, residue management, crop diversification play role in regulating the emergence rate of *P. minor* (Om et al., 2004, Frank et al., 2007). Zero-till wheat after DSR or MTR recorded a lower density of *P. minor* compared to conventional-till wheat during initial years. The seed germination of *P. minor* is stimulated by light (Om et al., 2003). Crop residue retention on soil surface and minimum soil disturbance in ZTW might have limited solar radiation to enter the soil compared to CT system, resulting in reduced seed germination. However, the higher density of *P. minor* in ZTW preceded by DSR in the 3^rd^ year might be due to accumulation of a greater number of weed seeds on soil surface with time. SWI had the maximum density of *S. nigrum* probably due to lack of crop residue cover on soil surface and lower shading effect by crop owing to wider plant spacing. Higher density of *S. nigrum* in conventional till system due to favourable growth conditions, was also reported by Bilalis et al. (2001). ZTW after DSR system resulted in a significant reduction in emergence of *C. album* compared to SWI after SRI production system. A lower density of *C. album* in ZT wheat (Lowry et al., 2021), and higher in CT system have also been reported (Swanton et al., 1999, Shrestha et al., 2002, Mishra et al., 2019b). Seeds of many weed species including *C. album* require brief exposure to light to break seed dormancy and induce germination (Buhler, 1997). Tillage system and environmental conditions influence the phenology of *C. album* seedling emergence (Roman et al., 2000). CT system allows more light to penetrate in soil compared to ZT, resulting in higher germination of *C. album*.

Irrespective of tillage and crop establishment methods, total weed density in wheat increased progressively up till year 3 and started declining thereafter; however, trend of increase varied under different treatments. In ZTW preceded by DSR or MTR, increase was up to 4^th^ year, whereas in case of SWI and CTW, the weed density increased till 3^rd^ year only. Variation in increased pattern under different TCE methods was due to its differential effects on individual weed flora. The lower density in ZTW was due to mulching effect of anchored residues of the previous crops. SWI system of wheat establishment had the maximum weed density in all the years due to wider plant spacing that encouraged a greater number of weeds to emerge and grow. Bilalis et al. (2001) also reported a higher density of annual weeds in conventional till system due to favourable growth conditions created by soil tillage.

The maximum mean weed dry biomass (420 g m^–2^) in rice under ZTMTR-ZTW system was due to vigorous growth of weeds, especially *E. colona* and *L. parviflora* prevalent in this system. This system also favoured infestation of grassy weed *Cynodon dactylon* in year 4 and 5 of the experiment. *Cynodon dactylon* plants were not counted because of practical difficulty in counting individual plant. Instead, plants of this species were expressed in terms of biomass and added to total weed biomass. Higher dry matter accumulation by weeds due to their vigorous growth suppressed the rice growth and development, resulting in lower crop yield (Table 5). Earlier studies also reported a higher infestation of *C. dactylon* in ZT system in IGP (Kumar et al., 2013). Higher density and vigorous growth of weeds (Table 3) in wheat in SWI system due to wider spacing resulted in higher dry matter accumulation.

### 4.2 Weed seedbank

Soil weed seedbank is the major source of weeds that determines above-ground weed flora composition and density in agricultural fields. The seedbank comprises new weed seeds recently shed by plant and the older seeds already present in the soil for many years (Norris, 2007, Skuodienė et al., 2013). As most weed seeds are present in the upper soil layer inreduced/zero tillage systems (Tørresen et al., 2003, Nakamoto et al., 2006, Chauhan and Johnson, 2009b, Mishra and Singh, 2012, Nath et al., 2017), surface loaded weed seeds in zero-till system already germinated in 3–4 years in the field and exhausted the seedbank (Nandan et al., 2020) resulting in lower weed seedling emergence. Further, weed seeds lying on soil surface are more prone to predation (Crutchfield et al., 1986), resulting in a reduction in seedbank and viability. Crop residue mulch on soil surface in zero-tilled systems suppresses weed seedling emergence, delays the time of emergence, and allows crops to gain an advantage over weeds (Chauhan and Johnson, 2010). Tillage can stimulate some weed seeds to germinate and bury other seeds which can remain viable in the soil for many years. Many weed seedlings fail to emerge if weed seeds are placed deeply (Chauhan and Johnson, 2008). Higher relative abundance of *C. iria* under ZTDSR-ZTW from 0 to 15 cm soil depth (Fig. 2) is likely related to their relatively smaller seed size and lesser seed energy reserves, which failed to emerge when buried deeply by CT (Chauhan and Johnson, 2009a, Mishra and Singh, 2012). Weed species such as *C. iria*, *C. difformis*, *F. miliacea* and *L. chinensis* could not emerge from a depth greater than 0.5 cm (Chauhan and Johnson, 2009b, Chauhan and Johnson, 2010). Due to lack of soil disturbance, ZT system accumulates weed seeds at soil surface (Hoffman et al., 1998, Chauhan et al., 2006a) and favours those species that can germinate from shallow depths or from within the surface residue layer (Barberi, Cascio, B, 2001) or that require light to germinate (El Titi, 2003, Chauhan and Johnson, 2009a).

In the present study (Fig. 2), compared to *C. iria* (28%), a relatively higher proportion of grassy (38%), and broad-leaved weed seeds (36%) were located in deeper soil layer. This might be due to relatively larger seed size, especially for broad-leaved weeds. Relative abundance of broad-leaved weeds in the upper soil layer was however higher under puddled transplanted rice-conventional till wheat (SRI-SWI, RPTR-BCW, LPTR-CTW) systems. Broad-leaved weed seeds in these systems might have been brought up to surface by repeated tillage operations in both rice and wheat. In contrast to *C. iria* and grassy weeds, total broad-leaved weed seeds were almost equally distributed at 15–30 cm soil depths in zero-till production systems compared to other TCE methods. Variations in tillage and crop establishment practices can modify the vertical distribution of weed seeds in soil (Chauhan et al., 2006b, Singh et al., 2015), influencing the seed germination and seedling emergence and species composition of weeds in field. Higher weed seed densities in upper soil layers in zero-till systems may be the result of reduced herbicides availability because of adsorption to near-surface organic matter (Isensee and Sadeghi, 1994). Seed size and soil type (Carter and Ivany, 2006) can also influence weed seedbank composition in soil. Adoption of zero-tillage and surface residue retention reduced the weed density and seedbank after 3–4 years and can favour crop growth. Non-disturbance of surface soil also prevents the buried weed seeds to germinate.

### 4.3 Yield and system productivity

**Fig. 3:**
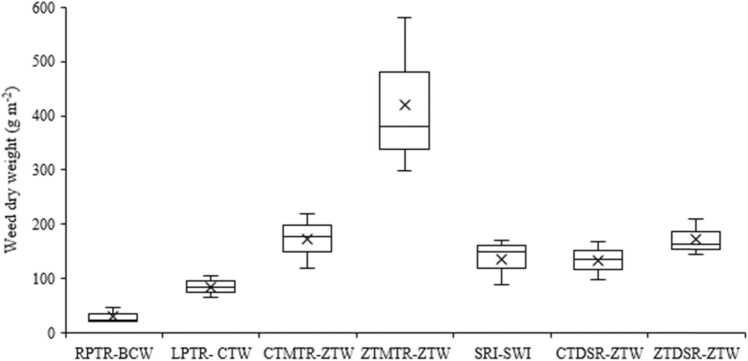
Box and whisker plot of mean total weed dry weight in rice at 75 days after sowing; ‘X′ represents mean. [RPTR: Puddled random transplanted rice; BCW: Broadcast wheat; LPTR: Puddled line transplanted rice; CTW: Conventional-till wheat; CTMTR: CT machine transplanted rice; ZTW: Zero-till wheat; ZTMTR: Zero-till MTR; SRI: System of rice intensification; SWI: System of wheat intensification; DSR: Direct-seeded rice].

The lowest rice grain yield recorded with ZTMTR was probably due to complete removal of mungbean biomass from field before mechanical transplanting, and infestation of weeds, especially *C. iria,* and *E. colona* (Table 2), and *C. dactylon* (data not presented), and higher weed biomass (Fig. 3). Removal of previous crop biomass from ZTMTR is necessary for smooth running of rice transplanter in zero-till field. In our study, rice yields in LPTR and RPTR were the same after 5^th^ year cycle, which is in contrast to earlier findings of Awan et al. (2011) who reported a 45% yield increase in LPTR as compared to RPTR. Results of a meta-analysis also indicated a 12% yield reduction in DSR than that of PTR (Xu et al., 2019). The lower yield in DSR could be attributed to a smaller number of spikelets per panicle, higher spikelet sterility (Singh et al., 2020), and lower grain weight (Mishra et al., 2021). Although panicle number per m^–2^ was more in DSR than PTR (data not presented), this increase in panicle number was not sufficient to compensate the reduction in spikelet number per panicle in DSR (Xu et al., 2019). Heavy shading before heading reduces hull size in DSR due to its higher plant density and height than PTR (Yoshida, 1981). Yield penalty in DSR production system can also be attributed to early weed competition during vegetative growth stage than in PTR where 25 days old rice seedlings and flooding have a competitive advantage over initial weed growth.

**Fig. 4:**
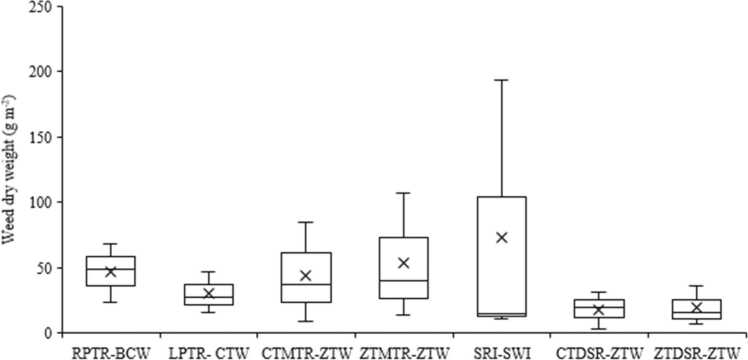
Box and whisker plot of mean total weed dry weight in wheat at 75 days after sowing; ‘X′ represents mean. [RPTR: Puddled random transplanted rice; BCW: Broadcast wheat; LPTR: Puddled line transplanted rice; CTW: Conventional-till wheat; CTMTR: CT machine transplanted rice; ZTW: Zero-till wheat; ZTMTR: Zero-till MTR; SRI: System of rice intensification; SWI: System of wheat intensification; DSR: Direct-seeded rice].

Wheat grain yield (4.83 Mg ha^–1^) in ZTDSR-ZTW was at par with that of CTDSR-ZTW and conventional system, but significantly higher by 11.5% over SRI-SWI (4.33 Mg ha^–1^). Conventional till drill-sown wheat produced a slightly higher (5%) yield than the broadcast sowing (Table 5). The study clearly shows that zero-till wheat after DSR proved beneficial in terms of grain yield. Better wheat yield after DSR is due to better soil aeration and structure which facilitates good plant growth and yield (Keil et al., 2017). In the present study, CT wheat yield after CT puddled rice was at par with ZT wheat after DSR, which was in contrast to previous studies (Gathala et al., 2011, Keil et al., 2015, Kumar et al., 2018, Kumari et al., 2011, Singh et al., 2020) where yield under CT wheat after puddled rice declined by 9–19%. It requires a thorough investigation at the farmer’s field with larger plot size so that the effect of smaller plots on sowing and irrigation operations can be minimised. There was no yield gain in SWI production system as also reported by Singh et al. (2020). Poor yield in SWI was due to lower plant density, a smaller number of spikes m^–2^ (data not included) and more weed competition (Fig. 4) during initial growth period due to wider space.

Due to continuous retention of anchored rice residue on soil surface in zero-tilled systems, a significantly (P < 0.05) higher density of armyworm was noticed in wheat in the 5^th^ year of experimentation compared to conventional systems (data not presented). Left-over rice residues acted as an alternate host for this insect. The larvae of *M. separata* damaged wheat leaves to varying degrees. Damage caused by this insect to grain yield of wheat under ZTDSR-ZTW was not visible (grain yield was higher compared to conventional system) because larval density (9 larvae m^–2^) was below the economic threshold level (ETL). Su and Lin (1987) reported ETL of *M. separata* in wheat as 14–34 larvae m^–2^. However, Kumar et al. (2022) indicated that *M. separata* may become an emerging threat to wheat production under zero-till systems in a long run, if not managed properly.

## 5 Conclusions

To address the problems of increasing water scarcity, deteriorating soil health, declining productivity, and climate change, sustainable intensification of rice-wheat cropping system with the inclusion of summer mungbean in eastern Indo-Gangetic Plains using resource conservation technologies are being developed and popularised. This study demonstrates that weed species are adapted to a specific establishment practice and soil disturbance levels, and any change in tillage and crop establishment practice leads to weed flora shift including the weed seedbank. Soil weed seedbank density and diversity are reliant on the soil disturbance level. Undisturbed soil system i.e., zero-till direct seeded rice – zero-till wheat, helped reduce the weed flora density and soil weed seedbank compared to conventional system. The results establish that the rice yields in triple zero-till based system (zero-till direct seeded rice with mungbean residue – zero-till wheat with rice residue – zero-till mungbean with wheat residue) were slightly lower than the conventional system, but because of the improvements in wheat yields, the system productivity did not decline. The zero-till direct seeded rice-based system also reduces overall weed problems with variable effects on individual weeds. Increasing infestation of armyworm (*Mythimna separata*) necessitates regular monitoring and management. However, declining groundwater resources confines the inclusion of the third crop of mungbean in rice-wheat rotation during summer season. Limited access to zero-till machinery by the small and marginal farmers in the eastern Indo-Gangetic Plains, severe weed problems and fear to lower crop yields, especially in zero-till direct seeded rice, crop residue burning and widespread use of crop residues for livestock feed are some of the major limitations in large-scale adoption of zero-till in the region. The information generated from the present study suggests that triple zero-till system would help in reducing the weed density and weed seedbank without affecting the system productivity in rice-wheat-mungbean crop rotation in the eastern Indo-Gangetic Plains.

## Declaration of Competing Interest

The authors declare that they have no known competing financial interests or personal relationships that could have appeared to influence the work reported in this paper.
