# Supplementary material for: Tillage and crop establishment effects on weeds and productivity of a rice-wheat-mungbean rotation
Source: Field Crops Res. 2022 Aug 1;284:108577. doi: 10.1016/j.fcr.2022.108577 (PMC9214547; doi:10.1016/j.fcr.2022.108577)
Supplement: Supplementary file 1 — Supplementary material [file mmc1.docx]

Supplementary Table 1: Effect of tillage and crop establishment methods on crop yields; mean values followed by different lower case letters within a column and different capital letters within a row or column are significantly different at P < 0.05. [RPTR: Puddled random transplanted rice; BCW: Broadcast wheat; LPTR: Puddled line transplanted rice; CTW: Conventional-till wheat; CTMTR: CT machine transplanted rice; ZTW: Zero-till wheat; ZTMTR: Zero-till MTR; SRI: System of rice intensification; SWI: System of wheat intensification; DSR: Direct-seeded rice].

| Treatments | Rice yield (Mg ha^-1^) | | | | | |
| --- | --- | --- | --- | --- | --- | --- |
|  | 2015 | 2016 | 2017 | 2018 | 2019 | Mean |
| RPTR-BCW | 7.15bcd | 6.24a | 5.7b | 7.25ab | 6.26a | 6.52AB |
| LPTR- CTW | 7.34abc | 6.48a | 5.9bb | 7.64a | 6.27a | 6.73A |
| CTMTR-ZTW | 7.51ab | 5.44abc | 5.69b | 6.49b | 5.28bc | 6.08AB |
| ZTMTR-ZTW | 7.68a | 5.82ab | 5.38b | 6.19b | 4.86c | 5.99B |
| SRI-SWI | 7.4ab | 5.83ab | 6.99a | 6.19b | 6.04ab | 6.49AB |
| CTDSR-ZTW | 6.94cd | 4.84bc | 6.94a | 7.29ab | 5.29bc | 6.26AB |
| ZTDSR-ZTW | 6.84d | 4.4c | 6.7a | 7.12ab | 5.46abc | 6.1AB |
| Mean | 7.27A | 5.58E | 6.19C | 6.88B | 5.64D |  |
| P-value (Y*T) | <0.01 | | | | |  |
|  | Wheat yield (Mg ha^-1^) | | | | | |
|  | 2015-16 | 2016-17 | 2017-18 | 2018-19 | 2019-20 | Mean |
| RPTR-BCW | 3.89b | 5.09bc | 3.94b | 4.6abc | 4.44ab | 4.39AB |
| LPTR- CTW | 4.45a | 5.53ab | 4.22ab | 5.13ab | 4.66ab | 4.8AB |
| CTMTR-ZTW | 3.94b | 5.45ab | 3.99b | 5.58a | 4.72a | 4.74AB |
| ZTMTR-ZTW | 4.23ab | 5.83a | 4.37a | 4.24bc | 4.43ab | 4.62AB |
| SRI-SWI | 4.22ab | 4.84c | 4.43a | 3.87c | 4.33b | 4.34B |
| CTDSR-ZTW | 4.16ab | 5.61ab | 4.22ab | 4.76abc | 4.71a | 4.69AB |
| ZTDSR-ZTW | 4.93a | 5.27abc | 4.43a | 4.95ab | 4.83a | 4.88A |
| Mean | 4.26D | 5.37A | 4.23E | 4.73B | 4.59C |  |
| P-value (Y*T) | <0.01 | | | | |  |
|  | Mungbean yield (Mg ha^-1^) | | | | | |
|  | 2016 | 2017 | 2018 | 2019 | 2020 | Mean |
| RPTR-BCW | 1.3a | 0.87c | 0.58c | 1.96a | 0.45c | 1.03B |
| LPTR- CTW | 1.19a | 0.99b | 0.76b | 2.3a | 0.52abc | 1.15AB |
| CTMTR-ZTW | 1.31a | 0.85c | 0.72b | 2.49a | 0.47c | 1.17A |
| ZTMTR-ZTW | 1.37a | 0.83c | 0.72b | 2.13a | 0.64a | 1.14AB |
| SRI-SWI | 1.36a | 1.01b | 0.7b | 2.09a | 0.57abc | 1.15AB |
| CTDSR-ZTW | 1.35a | 1.21a | 0.88a | 2.24a | 0.62ab | 1.26A |
| ZTDSR-ZTW | 1.26a | 1.01b | 0.84ab | 2.44a | 0.47bc | 1.20A |
| Mean | 1.31B | 0.97C | 0.74D | 2.24A | 0.53E |  |
| P-value (Y*T) | <0.01 | | | | |  |

Y: Year; T: Treatment
